# Health-related quality of life among caregivers of children with cerebral palsy in Sub-Saharan Africa: A systematic review and meta-analysis

**DOI:** 10.1371/journal.pmen.0000541

**Published:** 2026-04-07

**Authors:** Jenberu Mekurianew Kelkay, Desalegn Mitiku Kidie, Abraham Dessie Gessesse, Kaleab Tesfaye Tegegne, Henok Dessie Wubneh, Addisu Simachew Asgai, Deje Sendek Anteneh

**Affiliations:** 1 Department of Health Informatics, College of Health Sciences, Debark University, Debark, Ethiopia; 2 Department of pediatrics and child health nursing, college of health science, debark university, Debark, Ethiopia; 3 Department of pediatric and child health nursing, College of Health sciences, Woldia University, Woldia, Ethiopia; 4 Department of Public Health, College of Health Sciences, Debark University, Debark, Ethiopia; 5 School of Medicine, College of Medicine and Health Sciences, University of Gondar, Gondar, Ethiopia; 6 Department of Nursing, College of health sciences, Debark University, Debark, Ethiopia; 7 Department of Health Informatics, Institute of Public Health, College of Medicine and Health Sciences, University of Gondar, Gondar, Ethiopia; Hamdard University - Islamabad Campus, PAKISTAN

## Abstract

Cerebral palsy (CP) is a group of permanent movement and posture disorders that cause activity limitations, resulting from non-progressive disturbances in the developing fetal or infant brain. Caregivers of children with CP reported higher levels of stress, despair, and back discomfort. In Sub-Saharan Africa (SSA), caregivers of children with CP encounter complex, multidimensional challenges. While individual studies have examined the experiences of CP caregivers in SSA, no comprehensive review has yet synthesized evidence on their Health Related Quality of Life (HRQOL). Therefore, this SRMA aimed to synthesize evidence on the pooled mean estimate of HRQoL of caregivers of children with CP in Sub-Saharan Africa.A systematic search was conducted in PubMed, Scopus, Cochrane Library, and Google Scholar, supplemented by manual reference screening. Studies published between June 20 2000 and May 20 2025 in Sub-Saharan Africa, focusing on caregivers of children with cerebral palsy and their HRQoL, were included. Data were extracted using a standardized form, and quality was assessed via the Newcastle-Ottawa Scale. Random-effects meta-analysis was performed in STATA Version 17, with heterogeneity evaluated using Cochran’s Q and I^2^ statistics. Sensitivity analysis, subgroup analysis, and publication bias assessment (Begg’s and Egger’s tests) were conducted. The pooled mean of HRQoL of caregivers of cerebral palsy in Sub-Saharan Africa Countries is found to be 41.234 (95% CI: 23.315 to 59.154). The meta-analysis revealed significant regional disparities in HRQOL among CP caregivers in Sub-Saharan Africa. South Africa demonstrated superior and consistent outcomes (pooled mean: 67.52, I^2^ = 5.39%), attributable to robust healthcare systems.Caregivers of children with cerebral palsy in SSA experience moderate to low HRQoL, with significant regional disparities. Policy interventions such as expanding financial support, mental health programs, and community-based respite care are urgently needed. Strengthening caregiver training and regional disability policies can reduce disparities. Sustained research is essential to develop scalable, culturally appropriate solutions for improving caregiver well-being across SSA.

## Introduction

The prevalence of Cerebral Palsy (CP) decreased to 1.6 per 1000 live births in high-income nations due to improvements in obstetric procedures and perinatal treatment, while the prevalence in low- and middle-income nations, on the other hand, is still much greater, at 3.4 per 1000 live births [1]. CP is a group of permanent movement and posture disorders that cause activity limitations, resulting from non-progressive disturbances in the developing fetal or infant brain [2]. In addition to motor difficulties, cerebral palsy is accompanied by a variety of related comorbidities [3]. These usually involve communication difficulties, cognitive impairments, and sensory abnormalities that have a major influence on day-to-day functioning [4]. This multifaceted symptomatology demands comprehensive, multidisciplinary care models to address the full spectrum of patient needs [5].

Current evidence emphasizes the link between extended caregiving responsibilities, unfulfilled support needs, and poor physical and mental health among family caregivers [6–8]. When compared to caregivers of children without cerebral palsy, caregivers of children with CP reported higher levels of stress, hardship, and back discomfort [8]. The study identified socioeconomic status, child behavior, the extent of caregiving demands, and family dynamics as the principal factors influencing the health impacts on family caregivers [3]. Furthermore, among caregivers of children with cerebral palsy, favorable health outcomes were linked to a high reported level of social and marital support as well as the efficient application of stress management techniques [9]**.**

In Sub-Saharan Africa (SSA), caregivers of children with CP encounter complex, multidimensional challenges [10,11]. In the absence of formal support services, families in this area rely primarily on their own coping mechanisms and extended social networks to manage caregiving responsibilities [10,12]. While individual studies have examined the experiences of CP caregivers in SSA, no comprehensive review has yet synthesized evidence on their Health Related Quality of Life (HRQOL). Existing reviews primarily draw on research from high-income countries (HICs), with only one scoping review available for SSA. This significantly limits their relevance to the SSA context due to stark differences in socioeconomic conditions, cultural norms, and healthcare infrastructure [13–15].

Therefore, this systematic review and meta-analysis aimed to quantitatively synthesize evidence on the HRQoL of caregivers of children with cerebral palsy in Sub-Saharan Africa. By establishing the first pooled mean estimate of caregivers HRQoL for the country, this study addresses a critical evidence gap in low-resource settings where caregiving burdens are exacerbated by limited support systems. Preliminary searches confirmed no prior pooled mean SRMA had this population’s HRQoL outcomes in the SSA context, underscoring the novelty of this work for grading targeted interventions and policy development.

## Methods

We systematically searched for relevant articles from electronic databases directly related to the subject matter under review. The databases included PubMed, Scopus, Cochrane Library, and Google Scholar, along with manual searching of reference lists from eligible studies. Additionally, a supplemental search was performed by reviewing the reference lists of all included articles to ensure comprehensive coverage and reduce the risk of missing key studies.

A combination of Boolean operators “AND” and “OR” was applied to construct the search strategy. The final search was conducted up to 20 May 2025 using the following search terms:(“Cerebral Palsy” OR “CP” OR “Children with Cerebral Palsy”) AND(“Caregivers” OR “Parents” OR “Mothers” OR “Family caregivers”) AND(“Health-Related Quality of Life” OR “HRQoL” OR “Quality of Life”) AND(“Sub-Saharan Africa”)

To frame the research question and guide the review process, the COCOPOP framework was used as follows:

**Condition:** Caregiving for children with cerebral palsy

**Context:** Sub-Saharan Africa

**Outcome:** Health-Related Quality of Life (HRQoL)

**Population:** Caregivers (including parents, mothers, and family members) of children with cerebral palsy

### Eligibility criteria

Inclusion Criteria: Cross-sectional study designs published in English between January 1, 2000, and May 20 2025 and conducted exclusively in Sub-Saharan Africa. The search was limited to studies published from the year 2000 onward because the use and validation of standardized HRQoL instruments became more consistent after this period, and earlier studies were scarce and methodologically incompatible with contemporary HRQoL measurement standards. Although the initial manuscript was prepared earlier in 2025, the search was updated on 20 May 2025 during the revision process to ensure inclusion of the most recent studies. Although we initially planned to include studies published in English, our comprehensive search did not identify any relevant studies published in other languages. Therefore, no language-based exclusions were applied in practice, and the final set of included studies were all in English. This reduces the risk of language bias in our review.

### Exclusion criteria

Studies that were available only as abstracts or conducted in continents other than Africa were excluded.

### Data collection

The investigator developed a structured data extraction form using Microsoft Excel, guided by findings from the pilot review. Data extraction and calibration of the included studies were carried out accordingly. Two independent reviewers (DSA and KTT) extracted data from the eligible studies to assess study quality and synthesize findings. Any disagreements were resolved through discussion with a third reviewer (ADG). Extracted information included author, country, year of publication, study location, target population, details of exposure, standard deviation, sampling method, study design, outcome measures, risk of bias assessment, standard error, sample size, and effect size estimates.

### Data items of outcome

The outcome domain and its definition are as follows: Health-Related Quality of Life (HRQoL) refers to the perceived physical, psychological, and social well-being of caregivers of children with cerebral palsy [16]. It encompasses the impact of caregiving on their daily functioning, emotional state, social relationships, and overall quality of life.

### Quality assessment

Methodological quality and risk of bias were assessed using the **modified Newcastle–Ottawa Scale (NOS) for cross-sectional studies**, a validated tool for observational research [17]. Each study was independently assessed by two reviewers. Studies scoring **≥7 points** on the modified NOS were considered to be of adequate methodological quality. To ensure consistency and reliability, a quality control assessment was independently performed by three reviewers (**JMK, DSA, and KTT**). Discrepancies were resolved through consensus.

### Data analysis

All meta- analyses were performed using STATA version 17. We employed random effect model, because of presence of heterogeneity with in the studies. The summery of effect size for each study was calculated using the mean with 95% confidence interval. Statistical heterogeneity was assessed using cochran’s Q statistical test of and I^2^ statistics test to quantify the level of heterogeneity between studies. We considered I^2^ value greater than 50% to be indicative of substantial heterogeneity [18]. Sensitivity analysis wa**s** conducted using leave -out -one study meta -analysis method to evaluate the influence of each stud on the pooled effect size [3].

### Subgroup and meta-regression analysis

Subgroup analyses and meta-regression were conducted to explore potential sources of heterogeneity and assess the impact of study-level covariates on pooled effect sizes. Covariates were considered statistically significant if the p-value was < 0.05.

### Assessment of reporting bias

Publication bias was evaluated using **Begg’s funnel plot** and **Egger’s regression test** [19]. When required, corresponding authors of included studies were contacted via email to obtain missing or unclear methodological information.

## Results

**Fig 1:**
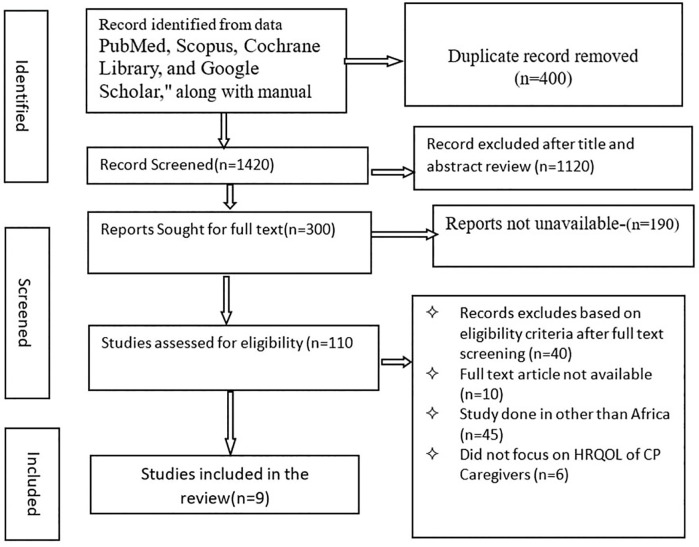
Flow Chart of Study Search Results Adapted from the PRISMA Statement to examine HRQoL of caregivers of CP: A SRMA of in Sub Saharan Africa, May 20 2025.

We have identified 1820 studies concerning about health related quality of life of caregivers of CP in Sub Saharan Africa (Table 1). After removal of duplications and application of inclusion /exclusion criteria, a total of 9 studies were identified, including 931 participants. Six studies were excluded because they did not focus on HRQoL of caregivers of children with cerebral palsy (n = 6) (Fig 1) [20]

**Table 1:** Characteristics of individual done on HRQoL of caregivers with CP in Sub Saharan Africa, 2025.

Author	Year of Publication	year of study	Region code name	region	study participants	Study design	Sample size	SD	Mean	Quality score (out of 10)
**Kassa et al.**	**2024**	**2022**	**East Africa**	**Ethiopia**	**Caregivers**	Cross sectional	324	**13.38**	**28.72**	**7**
**Fadwa et al**	**2019**	**2015**	**East Africa**	**Sudan**	**Caregivers**	Cross sectional	65	**8.85**	**8.85**	**8**
**Farah et al**	**2022**	**2019**	**East Africa**	**Kenya**	**Caregivers**	Cross sectional	50	**17.3**	**67.5**	**9**
**Namanja et al.**	**2022**	**2019**	**East Africa**	**Malawi**	**Caregivers**	Cross sectional	142	**11.43**	**52.2**	**6**
**Polack et al.**	**2018**	**2015**	**West Africa**	**Ghana**	**Caregivers**	Cross sectional	76	**15.41**	**12.5**	**9**
**Ferreira et al.**	**2018**	**2018**	**South Africa**	**South Africa**	**Caregivers**	Cross sectional	52	**18.8**	**66.23**	**8**
**Chidimma J. et al.**	**2018**	**2018**	**West Africa**	**Nigeria**	**Caregivers**	Cross sectional	78	**0.71**	**3.91**	**9**
**Dambi JM et al.**	**2015**	**2014**	**South Africa**	**Zimbabwe**	**Caregivers**	Cross sectional	48	**21.7**	**68.8**	**7**
**Gabriel et al.**	**2024**	**2022**	**West Africa**	**Nigeria**	**Caregivers**	Cross sectional	96	**18.42**	**57.63**	**9**

### Descriptive of original study

This research included, 9 cross-sectional studies (Table 1), published between 2015 and 2024. The sample size varied significantly, with the involving the largest 324 to the lowest 48. Overall, the meta-analysis combined data from 931 respondents to determine the overall mean of HRQOL in Sub Saharan Africa. The studies were conducted in different part of Sub Saharan countries: Ethiopia [21], South Africa [22], Nigeria [23–25], Kenya, Sudan [26], Malawi [27], Ghana [28] and Zimbabwe [29]. All of the studies were published. After reevaluating the articles, independent evaluators (JMK, DSA, and KTT) reached a consensus on studies that had previously been subject to debate during the screening process. All studies that achieved a quality score of at least six out of ten were included in the analysis.

### Meta-analysis

#### Mean of HRQOL.

In this meta-analysis, a random-effects model was used to estimate the pooled mean HRQoL among studies conducted in Sub-Saharan Africa (Fig 1). Because the included studies used different HRQoL measurement tools, substantial heterogeneity was expected. Subgroup-specific heterogeneity remained high within each instrument category: PedsQL-FIM (I² = 97.8%), SF-36 (I² = 99.6%), and WHOQOL-BREF (I² = 99.2%), all with p < 0.0001.

**Fig 2:**
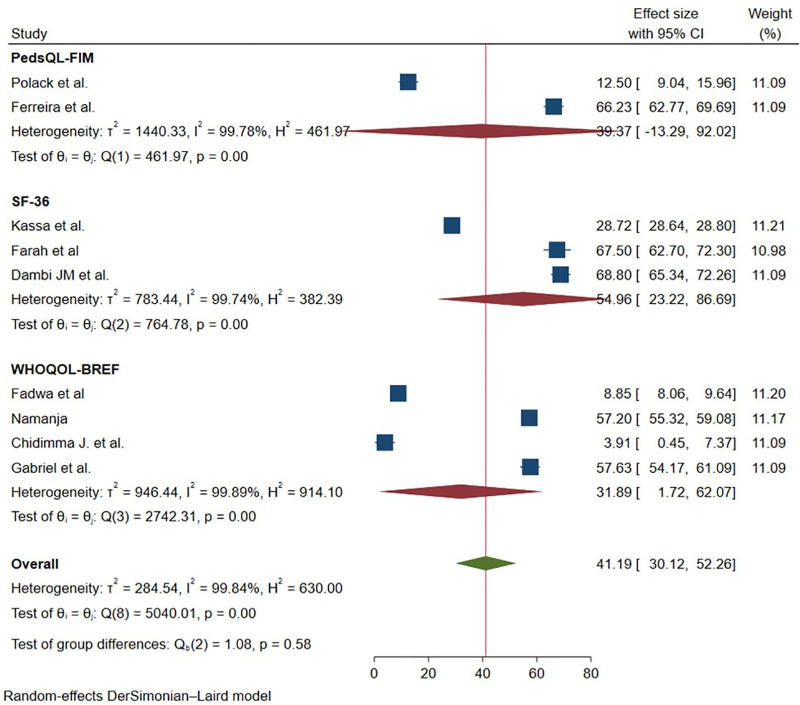
Forest plot of mean of HRQoL among caregivers of CP in Sab-Saharan Africa 2025.

In response to the high heterogeneity and the use of different HRQoL measurement tools, a subgroup analysis was performed based on the type of instrument used to avoid inappropriate cross-scale pooling. For studies using the PedsQL-FIM instrument, the pooled mean HRQoL score was 39.37 (95% CI: –13.29, 92.02), indicating substantial between-study variability. Studies that assessed HRQoL using the SF-36 reported a higher pooled mean score of 54.96 (95% CI: 23.22, 86.69). In contrast, studies employing the WHOQOL-BREF instrument yielded a pooled mean score of 31.89 (95% CI: 1.72, 62.07) (Fig 2). These instrument-specific pooled estimates provide a more accurate and interpretable summary of HRQoL outcomes, given the differences in scale structure and scoring across the tools used. To explore potential source of heterogeneity, a univariate meta- regression was conducted. The results showed that year of study (p = 0.011) and mean effect size (p = 0.000) and quality assessment score (p = 0.044) and) were significantly factors contributing to the observed heterogeneity (Table 2).

**Table 2:** Factors related to mean of HRQoL in Sub Saharan Africa 2025 (depend on meta-regression).

Variables	Coefficient	p-value	Confidence interval
**Mean**	1	0.000	0.9639636 1.036036
**Sample size**	-0.2364	0.231	-0.104636 .0104636
**Year of study**	0.00081	0.011	0.00051 0.00111

**Fig 3:**
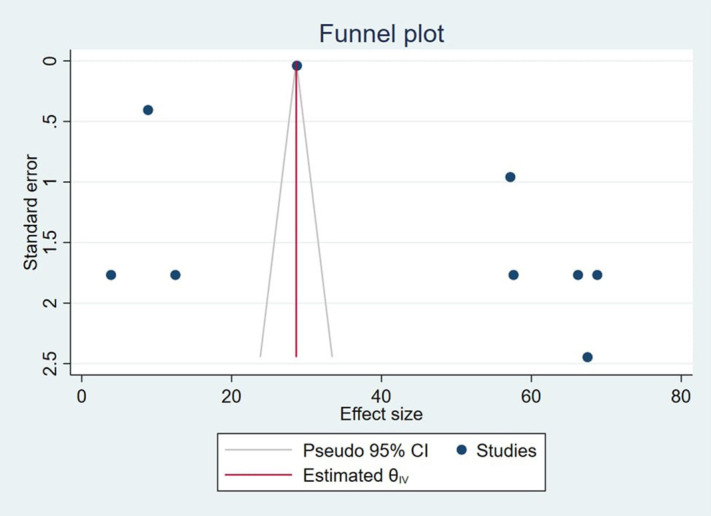
Funnel plot for detecting bias on HRQoL among caregivers of CP in Sab-Saharan Africa, 2025.

Therefore, Egger’s test for small-study effects was conducted using a random-effects model with year of publication, year of study, region, and sample size as moderators. The test revealed no significant evidence of publication bias (intercept = 11.49, SE = 29.32, z = 0.39, p = 0.70). Therefore, the results do not indicate the presence of small-study effects influencing the meta-analysis findings. The funnel plots also showed that the dots representing the studies, were asymmetrically indicated the presence of publication bias (Fig 3). Egger’s test indicated no statistically significant evidence of publication bias. However, visual inspection of the funnel plot suggested slight asymmetry, which can occasionally occur due to the small number of included studies or study heterogeneity. To account for this potential asymmetry, we performed a trim-and-fill sensitivity analysis, which did not substantially change the overall effect estimates, indicating that our findings are robust.

**Fig 4:**
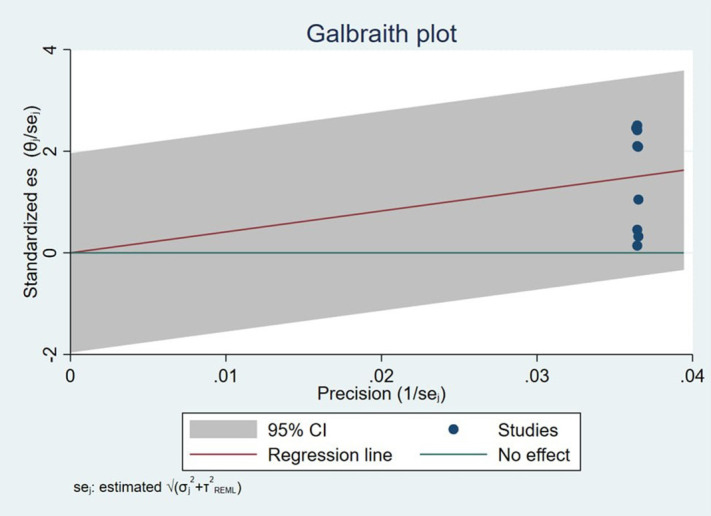
Galbraith plot showing heterogeneity bias on HRQoL among caregivers of CP in Sab-Saharan Africa, 2025.

The Galbraith plot shows (Fig 4) that all studies fall within the 95% confidence bands, with no significant outliers or asymmetry, suggesting low risk of publication bias and limited heterogeneity. These findings are consistent with the trim-and-fill analysis, which did not impute any missing studies.

### Sub group analysis

**Fig 5:**
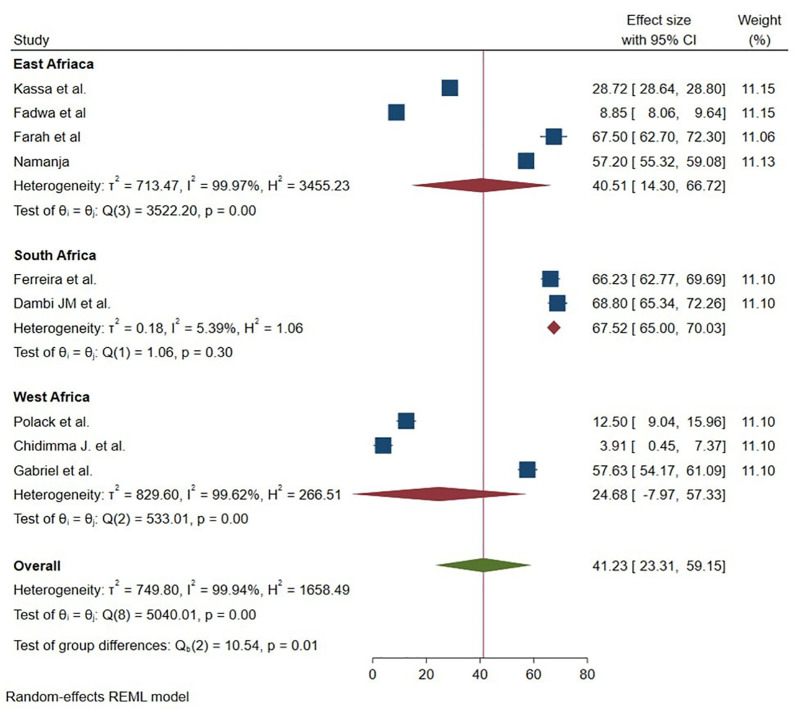
Graphical representation of sub group analysis by country of HRQoL among caregivers of CP in Sub Saharan Africa 2025.

**Fig 6:**
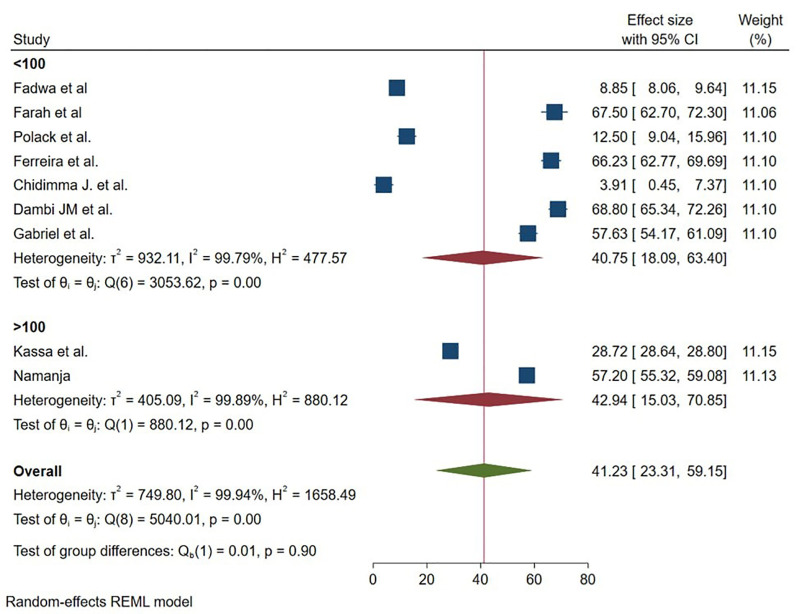
Graphical representation of sub group analysis by sample size of HRQoL among caregivers of CP in Sub Saharan Africa 2025.

Also, we performed subgroup analysis to explore sources of heterogeneity based on the continent where the studies conducted, sample size, year of study and year of publication. Accordingly, the highest mean was reported in the South Africa region with a mean of 67.52 (95% 65.00 -70.03) (Fig 5). Concerning sample size, the mean of HRQOL is found to nearly comparable in studies that had been included less than 100 respondents 40.75 (95% 18.09-63.40) and greater than 100 respondents 42.94 (95% 15.03 70.85) (Fig 6).

**Fig 7:**
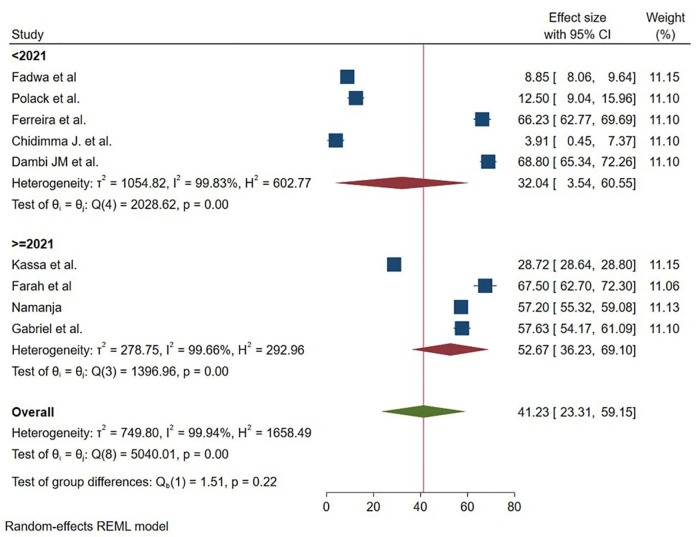
Graphical representation of sub group analysis by year of publication of HRQoL among caregivers of CP in Sub Saharan Africa 2025.

**Fig 8:**
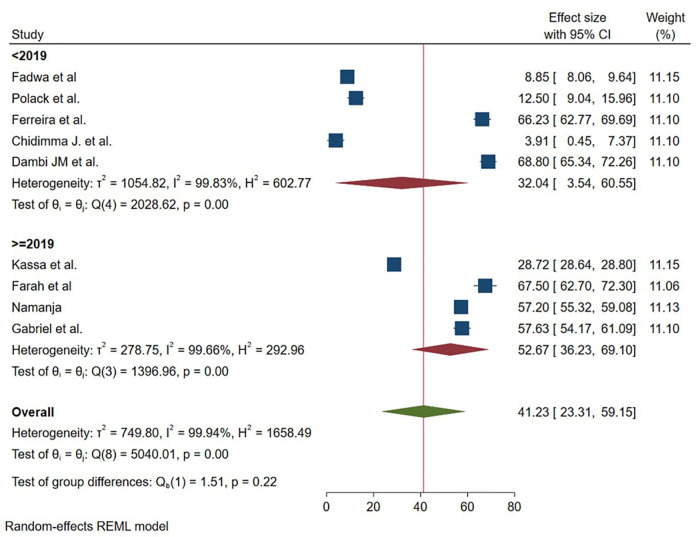
Graphical representation of sub group analysis by year of study of HRQoL among caregivers of CP in Sub Saharan Africa 2025.

Regarding year of publication of studies, the mean of HRQOL was less than in studies that had been done before 2021 32.04 (95% 3.54 60.55) as compared with those studies carried out after 2021 52.67 (95% 36.23 69.10) (Fig 7). Lastly year of studies, the mean of HRQOL was less than in studies that had conducted before 2019 32.04 (95% 3.54 60.55) as compared with those studies carried out after 2019 52.67 (95% 36.23 69.10) (Fig 8).

### Sensetivity analysis

**Fig 9:**
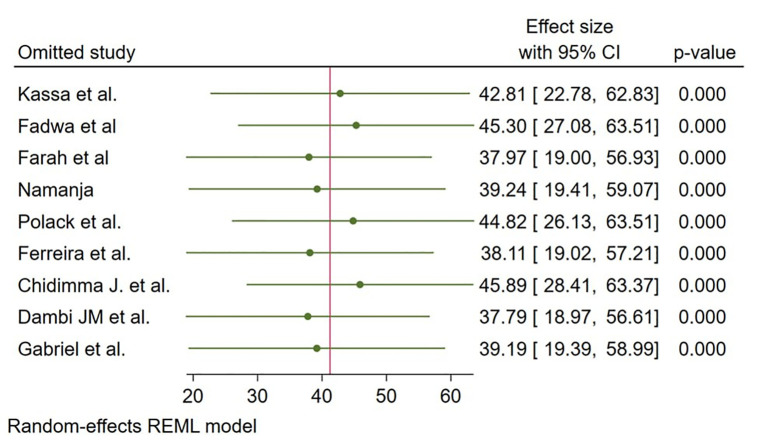
A leave- One -Study -Out Sensitivity Analysis of the HRQoL among caregivers of CP in Sub Saharan Africa 2025.

The sensitivity anaysis assessed the robustnes of the pooled mean effect size by systematically omitting stduies. Results showed any single study did not substanially alter the overall effect, which remained statistically significant (p-values = 0.000).The mean effect sizes ranged narrowly from 37.79 to 45.89, with overlapping 95% CLs, indicating consistent findings acorss studies. This suggets the meta-analysis conclusions are stable and not overly influnced by any single study (Fig 9).

## Discussion

This study aimed to revealed the pooled mean of HRQOL of caregivers with CP in Sub Saharan Africa countries. For studies using different HRQoL instruments, the pooled mean scores varied considerably. Studies employing the PedsQL-FIM reported a pooled mean of 39.37 (95% CI: –13.29, 92.02). Those using the SF-36 instrument had a higher pooled mean of 54.96 (95% CI: 23.22, 86.69), while studies utilizing the WHOQOL-BREF instrument reported a lower pooled mean of 31.89 (95% CI: 1.72, 62.07). The health-related quality of life (HRQOL) of caregivers for children with CP is recognized as a critical public health issue, particularly in low- and middle-income countries (LMICs), where 52.6% of burden [30].

However, in high-income settings such as Germany, the mean PedsQL-FIM HRQoL score for caregivers of children with autism was 40.15, which is very similar to the pooled mean obtained from studies in Sub-Saharan Africa using the same PedsQL-FIM instrument [31]. However, direct comparisons should be made cautiously due to difference in caregiving contexts, disability types and measurement tools. Caregiver stress in SSA and high-income countries like Germany is shaped by different social, economic, and cultural factors [32]. In SSA, financial hardship and resource scarcity are major stressors, but extended family and community support can help caregivers cope [32]. In contrast, caregivers in high-income countries may have better access to healthcare but face higher emotional and societal pressures due to less communal support [33]. These findings highlight the need for targeted interventions such as financial assistance, mental health support, and respite care to improve HRQOL for caregivers in SSA, while also emphasizing that even in high-resource settings caregivers of children with neurodevelopmental disorders remain vulnerable.

Studies employing the PedsQL-FIM reported a pooled mean HRQoL score of 39.37 (95% CI: –13.29 to 92.02), indicating a moderate-to-low quality of life among caregivers. Those using the SF-36 instrument showed a higher pooled mean of 54.96 (95% CI: 23.22 to 86.69), reflecting a moderate quality of life. In contrast, studies utilizing the WHOQOL-BREF reported a lower pooled mean of 31.89 (95% CI: 1.72 to 62.07), suggesting a low quality of life [21]. Overall, these instrument-specific findings indicate that caregivers of children with CP in Sub-Saharan Africa experience low to moderate HRQoL, depending on the measurement tool used. This significant caregiver burden stems from the lifelong care demands of CP, including mobility assistance, speech therapy, and seizure management, which often lead to fatigue, financial stress, and emotional distress. These challenges are further exacerbated by limited healthcare access across SSA, where scarce rehabilitation services and high out-of-pocket costs intensify caregiver strain. However, the HRQOL score may not be even lower due to potential protective factors such as extended family and community support systems, which are commonly found in many SSA cultures and may help buffer some of the psychological and practical stressors of caregiving [32].

The subgroup analysis of this study also showed that the mean of HRQOL of caregivers of CP varies across continent of SSA. The meta-analysis revealed significant regional disparities in HRQOL among CP caregivers in Sub-Saharan Africa. South Africa demonstrated superior and consistent outcomes (67.52, I² = 5.39%), attributable to robust healthcare systems, urbanization, and progressive policies like disability grants. In contrast, East Africa showed moderate but highly variable scores (40.51, I² = 99.97%), reflecting uneven healthcare access and socioeconomic diversity. West Africa reported the lowest HRQOL (24.68, I² = 99.62%), with extreme distress in some settings, linked to infrastructure gaps and economic hardship. These findings underscore the critical role of healthcare investment and policy frameworks in mitigating caregiver burden. Targeted interventions should prioritize expanding South Africa’s model of structured support to other regions, addressing rural-urban inequities in East Africa, and urgent poverty alleviation and anti-stigma measures in West Africa [34].

The meta-regression analysis revealed important temporal patterns in caregiver HRQOL outcomes. While we identified a statistically significant association between year of study and HRQOL scores (p = 0.011), the extremely small effect size suggests that improvements over time have been minimal. This finding implies that despite potential advances in healthcare systems and social support structures across Sub-Saharan Africa between the earliest (pre-2019) and most recent (post-2019) studies, these changes have translated to only negligible improvements in caregiver quality of life [35].

The stable baseline effect (p < 0.001) across all years of study underscores the persistent challenges faced by caregivers throughout the study period [36]. The non-significant association with sample size (p = 0.231) further strengthens our confidence in these temporal findings by ruling out study size as a confounding factor.

These results suggest that while some incremental progress may be occurring, the pace of improvement is insufficient to meaningfully alleviate caregiver burden. The minimal yearly change highlights the need for aggressive policy interventions and support programs specifically targeting caregivers of children with cerebral palsy, rather than relying on gradual systemic improvements [37]. Future research should investigate whether particular years or policy periods show more pronounced effects, which could help identify effective interventions worth scaling across the region.

### Limitations of the study

Geographic bias exists as most data came from select countries, leaving other SSA regions underrepresented. Second, reliance on cross-sectional studies limits causal inferences about HRQOL trends over time. Additionally, inconsistent HRQOL assessment methods across studies may have influenced pooled estimates.

## Conclusions

This meta-analysis reveals that caregivers of children with CP in Sub-Saharan Africa experiences a moderate to low health related quality of life, with significant regional disparities. South Africa demonstrates the highest HRQOL due to stronger healthcare systems and disability policies, while West Africa reports the lowest, reflecting severe economic and infrastructure challenges. Despite minor temporal improvements, HRQOL gains have been negligible over time, emphasizing that systemic progress alone is insufficient to alleviate caregiver burden.

Policy and programmatic interventions are urgently needed to improve the health-related quality of life of caregivers of children with CP in sub-Saharan Africa. Effective disability support models from South Africa should be adapted across the region, alongside targeted poverty alleviation and accessible mental health services in high-burden areas. Strengthening community-based respite care and caregiver training programs is essential to prevent burnout and leverage family support networks. Continued investment in longitudinal research is critical to inform culturally appropriate, scalable interventions.

## Supporting information

S1 Data Dataset used for meta-analysis. (CSV)

S1 Checklist PRISMA 2020 checklist. From: Page MJ, McKenzie JE, Bossuyt PM, Boutron I, Hoffmann TC, Mulrow CD, et al. The PRISMA 2020 statement: an updated guideline for reporting systematic reviews. BMJ 2021;372:n71. doi: 10.1136/bmj.n71. This work is licensed under CC BY 4.0. To view a copy of this license, visit https://creativecommons.org/licenses/by/4.0/.
(DOCX)

## References

[1] McIntyre S, Goldsmith S, Webb A, Ehlinger V, Hollung SJ, McConnell K, et al. Global prevalence of cerebral palsy: A systematic analysis. Dev Med Child Neurol. 2022;64(12):1494–506. doi: 10.1111/dmcn.15346 35952356 PMC9804547

[2] Bax M, Goldstein M, Rosenbaum P, Leviton A, Paneth N, Dan B, et al. Proposed definition and classification of cerebral palsy, April 2005. Dev Med Child Neurol. 2005;47(8):571–6. doi: 10.1017/s001216220500112x 16108461

[3] Markos M, Kefyalew B, Tesfaye HB. Pooled prevalence of blindness in Ethiopia: a systematic review and meta-analysis. BMJ Open Ophthalmol. 2022;7(1):e000949. doi: 10.1136/bmjophth-2021-000949 36161856 PMC9171275

[4] Fonzi V, Sheriff B, Dalglish S, Anum A, Dwomo Agyei E, Diggs D, et al. The multifaceted care-seeking practices among caregivers of children with cerebral palsy: Perspectives from mothers and providers in Ghana. PLoS One. 2021;16(10):e0258650. doi: 10.1371/journal.pone.0258650 34705843 PMC8550440

[5] Li X, Einfeld S, Stancliffe R, Hodge A. Executive function is associated with behaviour problems in children and adolescents with cerebral palsy and intellectual disability. J Intellect Dev Disabil. 2025;50(3):316–29. doi: 10.3109/13668250.2024.2446215 39819150

[6] Bearden DR, Monokwane B, Khurana E, Baier J, Baranov E, Westmoreland K, et al. Pediatric cerebral palsy in Botswana: etiology, outcomes, and comorbidities. Ped Neurol. 2016;59:23–9. 10.1016/j.pediatrneurol.2016.03.002 PMC4912921 27114082

[7] Davis E, Shelly A, Waters E, Boyd R, Cook K, Davern M, et al. The impact of caring for a child with cerebral palsy: quality of life for mothers and fathers. Child Care Health Dev. 2010;36(1):63–73. doi: 10.1111/j.1365-2214.2009.00989.x 19702639

[8] Boixadós Anglès M, Muñoz Marrón E, Redolar Ripoll DA, Guillamon Cano N, Gómez Zúñiga B, Hernàndez Encuentra E, et al. Impact of caring for a child with cerebral palsy on the quality of life of parents: a systematic review of the literature. 2013.

[9] Raina P, O’Donnell M, Rosenbaum P, Brehaut J, Walter SD, Russell D, et al. The health and well-being of caregivers of children with cerebral palsy. Pediatrics. 2005;115(6):e626-36. doi: 10.1542/peds.2004-1689 15930188

[10] Olawale OA, Deih AN, Yaadar RK. Psychological impact of cerebral palsy on families: The African perspective. J Neurosci Rural Pract. 2013;4(2):159–63. doi: 10.4103/0976-3147.112752 23914092 PMC3724294

[11] Melak M, Fakolade A, Mekonnen S, Baraki A, Ross-White A, Batorowicz B. The state of evidence on the health outcomes and support needs of family caregivers of children with Cerebral Palsy in Sub-Saharan Africa: a scoping review. Disabil Rehabil. 2025;47(21):5435–50. doi: 10.1080/09638288.2025.2472984 40055874

[12] Hamzat TK, Mordi EL. Impact of caring for children with cerebral palsy on the general health of their caregivers in an African community. Int J Rehabil Res. 2007;30(3):191–4. doi: 10.1097/MRR.0b013e3281e5af46 17762763

[13] Dlamini MD, Chang Y-J, Nguyen TTB. Caregivers’ experiences of having a child with cerebral palsy. A meta-synthesis. J Pediatr Nurs. 2023;73:157–68. doi: 10.1016/j.pedn.2023.08.026 37690430

[14] Elangkovan IT, Shorey S. Experiences and Needs of Parents Caring for Children with Cerebral Palsy: A Systematic Review. J Dev Behav Pediatr. 2020;41(9):730–9. doi: 10.1097/DBP.0000000000000880 33136702

[15] Smith M, Blamires J. Mothers’ experience of having a child with cerebral palsy. A systematic review. J Pediatr Nurs. 2022;64:64–73. doi: 10.1016/j.pedn.2022.01.014 35158294

[16] Bjornson KF, McLaughlin JF. The measurement of health-related quality of life (HRQL) in children with cerebral palsy. Eur J Neurol. 2001;8 Suppl 5:183–93. doi: 10.1046/j.1468-1331.2001.00051.x 11851747

[17] Wubante SM, Tegegne MD, Melaku MS, Walle AD, Demsash addisalem workie. Knowledge sharing practice and its associated factors among health professionals in Ethiopia: Systematic review and meta-analysis. Inform Med Unlocked. 2022;31:100967. doi: 10.1016/j.imu.2022.100967

[18] Borenstein M, Higgins JPT, Hedges LV, Rothstein HR. Basics of meta-analysis: I2 is not an absolute measure of heterogeneity. Res Synth Methods. 2017;8(1):5–18. doi: 10.1002/jrsm.1230 28058794

[19] Lin L, Chu H, Murad MH, Hong C, Qu Z, Cole SR, et al. Empirical Comparison of Publication Bias Tests in Meta-Analysis. J Gen Intern Med. 2018;33(8):1260–7. doi: 10.1007/s11606-018-4425-7 29663281 PMC6082203

[20] Basha GK, van Heerden HJ. Profile and opinion of people with disability with respect to adapted physical activity participation in Ethiopia. Afr J Disabil. 2020;9:657. doi: 10.4102/ajod.v9i0.657 33102185 PMC7564746

[21] Kassa T, Tadese H, Eriku GA, Abich Y, Fentanew M. Health-related quality of life and associated factors among primary caregivers of children with cerebral palsy, in Bahir Dar and Gondar cities, Ethiopia, 2022. PLoS One. 2024;19(4):e0301050. doi: 10.1371/journal.pone.0301050 38687770 PMC11060524

[22] Le Roux R. Caregivers? perceptions of caregiver burden, quality of life and support needs in caring for a child with cerebral palsy with feeding and/or swallowing difficulties within the context of the Western Cape, South Africa. 2023.

[23] Umar AB, Yakasai AM, Danazumi MS, Shehu UT, Badaru UM, Kaka B. Assessment of family needs of children with cerebral palsy in Northern-Nigeria: A cross-sectional study. J Pediatr Rehabil Med. 2021;14(2):265–74. 34092657 10.3233/PRM-200696

[24] Fatudimu MB, Hamzat TK, Akinyinka OO. Comparative quality of life of Nigerian caregivers of children with cerebral palsy. Int J Ther Rehabil. 2013;20(3):131–5. doi: 10.12968/ijtr.2013.20.3.131

[25] Ahanotu CJ, Ibïkunle PO, Hammed AI. Burden of caregiving, social support and quality of life of informal caregivers of patients with cerebral palsy. Turk J Kinesiol. 2018;4(2):58–64. doi: 10.31459/turkjkin.418491

[26] Mohammed FMS, Ali SM, Mustafa MAA. Quality of life of cerebral palsy patients and their caregivers: A cross sectional study in a rehabilitation center Khartoum-Sudan (2014 - 2015). J Neurosci Rural Pract. 2015;7(3):355–61. doi: 10.4103/0976-3147.182778 27365951 PMC4898102

[27] Namanja A, Phiri VS. Quality of life of primary caregivers of children living with cerebral palsy at two clinics in Blantyre, Malawi. Malawi Med J. 2022;34(3):176–83. doi: 10.4314/mmj.v34i3.6 36406099 PMC9641608

[28] Polack S, Adams M, O’banion D, Baltussen M, Asante S, Kerac M, et al. Children with cerebral palsy in Ghana: malnutrition, feeding challenges, and caregiver quality of life. Dev Med Child Neurol. 2018;60(9):914–21. doi: 10.1111/dmcn.13797 29736993

[29] Dambi J, Makotore F, Kaseke F. The impact of caregiving a child with cancer: A cross sectional study of experiences of Zimbabwean caregivers. J Palliat Care Med. 2015;5(05):10.4172.

[30] Power R, King C, Muhit M, Heanoy E, Galea C, Jones C, et al. Health-related quality of life of children and adolescents with cerebral palsy in low- and middle-income countries: a systematic review. Dev Med Child Neurol. 2018;60(5):469–79. doi: 10.1111/dmcn.13681 29405292

[31] Dückert S, Bart S, Gewohn P, König H, Schöttle D, Konnopka A, et al. Health-related quality of life in family caregivers of autistic adults. Front Psychiatry. 2023;14:1290407. doi: 10.3389/fpsyt.2023.1290407 38193135 PMC10773769

[32] Chatterji S, Aldwin C, Kowal P, Small J. Aging and HIV-related caregiving in Sub-Saharan Africa: A Social Ecological Approach. Gerontologist. 2019;59(3). 10.1093/geront/gnx159 PMC6524476 29045750

[33] Zhang Y, Xiao L, Wang J. Chinese diaspora caregivers’ experiences in dementia care in high-income countries: A systematic review. Dementia (London). 2023;22(5):1115–37. doi: 10.1177/14713012231169830 37072899 PMC10262336

[34] Madzimbe P, Maart S, Corten L, Dambi J. Participation of fathers and siblings in home rehabilitation programmes for children with neuro-developmental delay: a scoping review. BMC Pediatr. 2024;24(1):659. doi: 10.1186/s12887-024-05119-w 39402501 PMC11472531

[35] Andualem A, Gelaye H. Quality of life and associated factors among family caregivers of individuals with psychiatric illness at DRH, South Wollo, Ethiopia, 2020. Sci Rep. 2022;12. 10.1038/s41598-022-22015-4 PMC9633784 36329187

[36] Akbari M, Alavi M, Irajpour A, Maghsoudi J. Challenges of Family Caregivers of Patients with Mental Disorders in Iran: A Narrative Review. Iran J Nurs Midwifery Res. 2018;23(5):329–37. doi: 10.4103/ijnmr.IJNMR_122_17 30186336 PMC6111657

[37] Vadivelan K, Sekar P, Sruthi SS, Gopichandran V. Burden of caregivers of children with cerebral palsy: an intersectional analysis of gender, poverty, stigma, and public policy. BMC Public Health. 2020;20(1):645. doi: 10.1186/s12889-020-08808-0 32384875 PMC7206712

